# Supplementary material for: Assessing Women’s Menstruation Concerns and Experiences in Rural India: Development and Validation of a Menstrual Insecurity Measure
Source: Int J Environ Res Public Health. 2020 May 15;17(10):3468. doi: 10.3390/ijerph17103468 (PMC7277189; doi:10.3390/ijerph17103468)
Supplement: Supplementary file 1 [file ijerph-17-03468-s001.pdf]

Supplemental Materials

Assessing Women's Menstruation Concerns and Experiences in Rural India: Development and Validation of a Menstrual Insecurity Measure

Bethany A. Caruso [1,*], Gerard Portela [2], Shauna McManus [3] and Thomas Clasen [4]

1 Hubert Department of Global Health, Rollins School of Public Health, Emory University, Atlanta, GA 30322, USA
2 Department of Epidemiology, Rollins School of Public Health, Emory University, Atlanta, GA 30322, USA; gerard.portela@emory.edu
3 Department of Biostatistics and Bioinformatics, Rollins School of Public Health, Emory University, Atlanta, GA 30322, USA; shauna.mcmanus@emory.edu
4 Gangarosa Department of Environmental Health, Rollins School of Public Health, Emory University, Atlanta, GA 30322, USA; thomas.f.clasen@emory.edu
* Correspondence: bethany.caruso@emory.edu

Received: 31 March; Accepted: 9 May; Published: date

Abstract: Qualitative research has documented menstruator's challenges, particularly in water and sanitation poor environments, but quantitative assessment is limited. We created and validated a culturally-grounded measure of Menstrual Insecurity to assess women's menstruation-related concerns and negative experiences. With cross-sectional data from 878 menstruating women in rural Odisha, India, we carried out Exploratory (EFA) and Confirmatory (CFA) Factor Analyses to reduce a 40-item pool and identify and confirm the scale factor structure. A 19-item, five factor model best fit the data (EFA: root mean square error of approximation (RMSEA) = 0.027; comparative fit index (CFI) = 0. 0.994; Tucker-Lewis index (TLI) = 0.989; CFA: RMSEA = 0.058; CFI = 0.937; TLI = 0.925). Sub-scales included: Management, Menstrual Cycle Concerns, Symptoms, Restrictions, and Menstruation-Related Bodily Concerns. Those without access to a functional latrine, enclosed bathing space, water source within their compound, or who used reusable cloth had significantly higher overall Menstrual Insecurity scores (greater insecurity) than those with these facilities or using disposable pads. Post-hoc exploratory analysis found that women reporting experiencing tension at menstrual onset or difficulty doing work had significantly higher Menstrual Insecurity scores. This validated tool is useful for measuring Menstrual Insecurity, assessing health inequities and correlates of Menstrual Insecurity, and informing program design.

Keywords: gender; security; factor analysis; management; restriction; pain; menstrual health

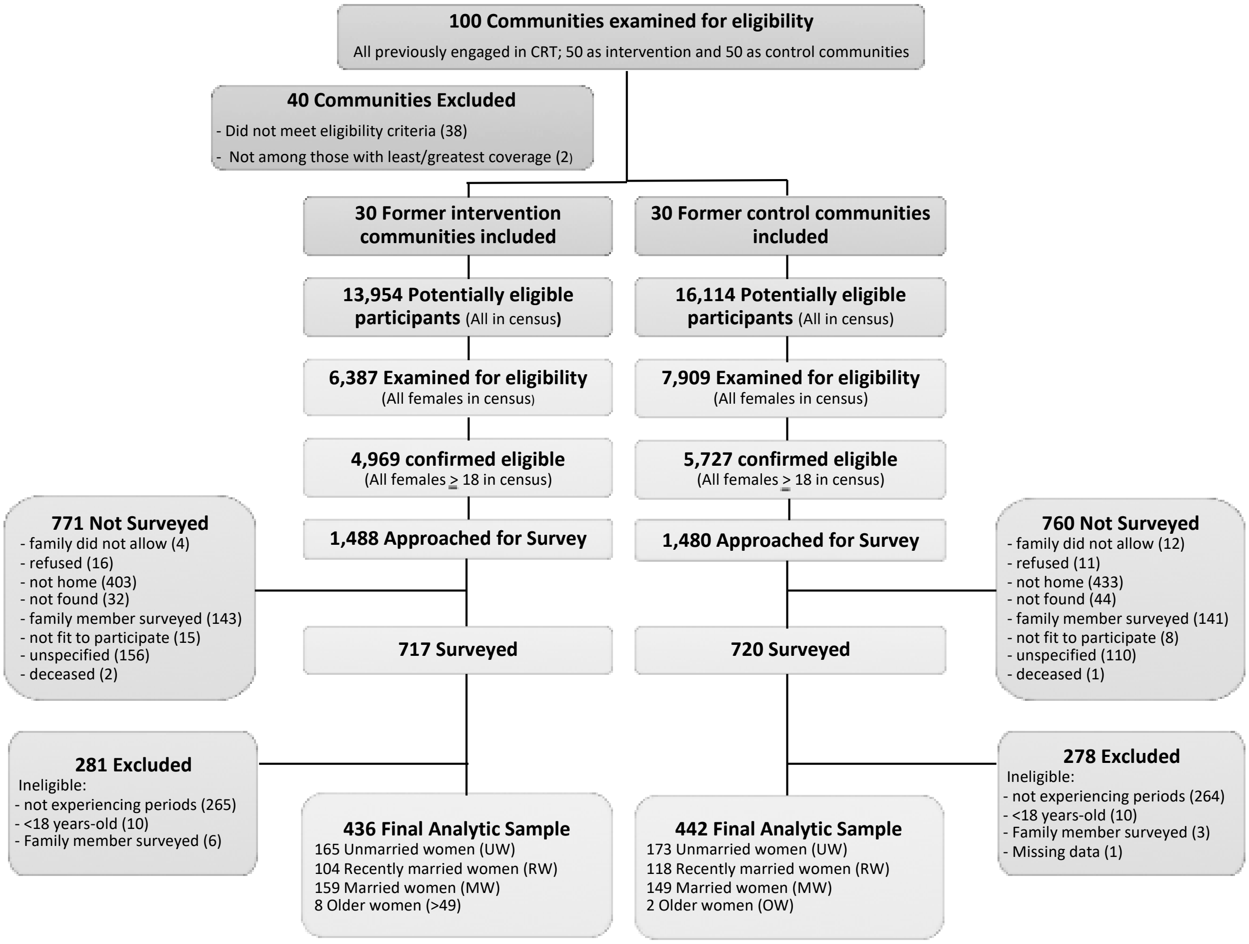

Figure S1. Flow diagram illustrating community and individual eligibility, exclusion, non-participation, and inclusion in EFA/CFA.

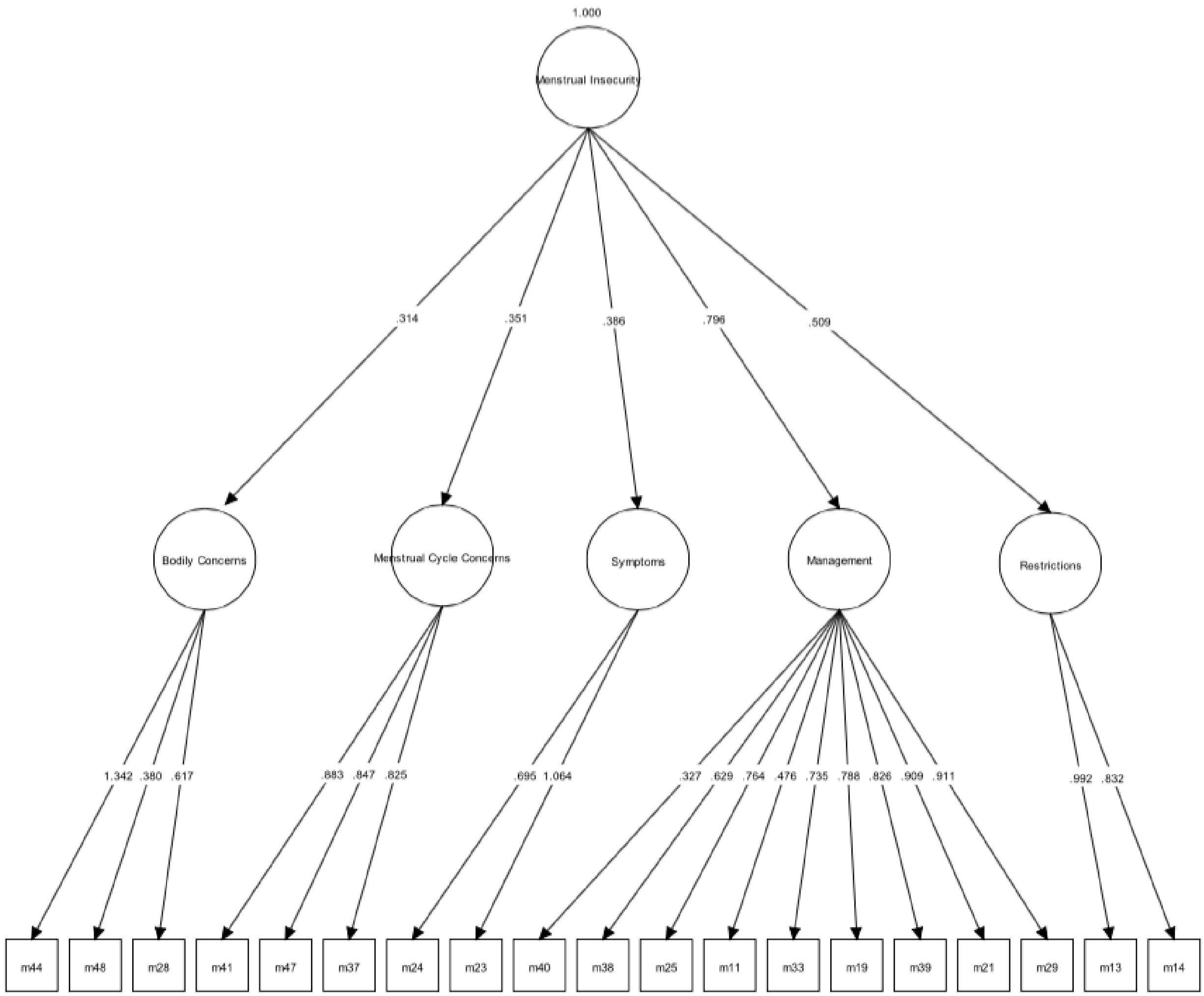

Figure S2. Diagram of Hierarchical Confirmatory Factor Analysis Model.

Table S1. Table of Survey Items Grouped by Four Emergent Themes and Post-EFA/CFA Results.

Emergent Themes and Associated Survey Items		EFA/CFA Result
**Management**		
M11	Could not get the material I needed, like cotton cloth or sanitary pad	In hypothesized *Management* Factor
M19	Experienced difficulty bathing during menstruation	In hypothesized *Management* Factor
M20	Got blood stains or leaks on my clothes	Dropped
M21	Had difficulty finding a place to wash cloth	In hypothesized *Management* Factor
M24	Had difficulty finding a suitable place to dry my menstrual cloth	In hypothesized *Management* Factor
M27	Had a lot of cleaning work to do because of menstruation	Dropped
M29	Had difficulty finding a place to change menstrual materials (cloth / pad)	In hypothesized *Management* Factor
M31	Had difficulty fetching water for menstruation related needs	Dropped
M33	Had difficulty finding a place to dispose of cloth or pad	In hypothesized *Management* Factor
M39	Had difficulty finding a place to store menstrual cloth or pads	In hypothesized *Management* Factor
M43	Had to wear cloth that was not fully dry after washing	Dropped
M48	Worried that my cloth, napkin or my body smelled	In *Menstruation-Related Bodily Concerns* Factor
**Restrictions or challenges to normal activities**		
M10	Avoided leaving the house during menstruation	Dropped
M12	Did not feel like eating during menstruation	Dropped
M13	Worry about not being able to participate in religious activities	In hypothesized *Restrictions* Factor
M14	Could not touch certain things, which created difficulties for me	In hypothesized *Restrictions* Factor
M17	Did not feel like interacting with others during menstruation	Dropped
M30	Had difficulty doing my regular work during menstruation	Dropped
M32	Had difficulty urinating while menstruating	Dropped
M34	Had difficulty walking during menstruation	Dropped
M35	Was forced to conform to a restriction that I do not believe in	Dropped
M40	Had to stay separated at night from my normal bed	In *Management* Factor
M44	Had trouble doing my work because of wound from cloth/pad	In *Menstruation-Related Bodily Concerns* Factor
M46	Was prevented from going to certain places that I wanted to go to	Dropped
**Social Needs and Constraints**		
M15	Worried about needing other's support to get the cloth or sanitary pads I needed	Dropped
M36	Had people tease me because they knew I was menstruating	Dropped
M38	Had difficulty finding someone to help me with bathing and other menstruation-related needs at onset	In *Management* Factor
M42	Worried about being treated as untouchable by others	Dropped
M49	Worried that others knew I was menstruating	Dropped
**Well-Being**		
M16	Experienced headache during menstruation	Dropped
M18	Experienced burning and irritation in urinary tract during menstruation	Dropped
M22	Experienced heavy bleeding	Dropped
M23	Experienced pain in the hands or legs during menstruation	In *Symptoms* Factor
M24	Experienced stomach pains during menstruation	In *Symptoms* Factor
M26	Felt like vomiting during menstruation	Dropped
M28	Got wounds on inner thighs from cloth or belt	In *Menstruation-Related Bodily Concerns* Factor
M37	Worried that my cycle was irregular	In *Menstrual Cycle Concerns* Factor
M41	Worried about my health because of problems with my menstrual cycle	In *Menstrual Cycle Concerns* Factor
M45	Have a general feeling of tension at onset of menstruation	Dropped
M47	Worried about ability to become pregnant because of problems with my menstrual cycle	In *Menstrual Cycle Concerns* Factor

Table S2. Menstrual Insecurity Item frequencies among women in rural Orissa, India (n = 878)

	Full Sample (%)					
	Never	Sometimes	Often	Always	Skewness	Kurtosis
**Factor 1: Restrictions**						
Could not touch certain things, which created difficulties for me	297 (34)	276 (31)	129 (15)	176 (20)	0.45	1.16
Worry about not being able to participate in religious activities	241 (27)	261 (30)	121 (25)	255 (29)	0.16	1.46
**Factor 2: Management**						
Had difficulty finding a place to change menstrual materials (cloth or pad)	804 (92)	35 (4)	15 (2)	24 (3)	4.00	15.53
Had difficulty finding a place to wash cloth	717 (82)	51 (6)	40 (5)	70 (8)	2.18	3.23
Had difficulty finding a place to store menstrual cloth or pads	819 (93)	30 (3)	9 (1)	20 (2)	4.64	21.44
Experienced difficulty bathing during menstruation	676 (77)	103 (12)	43 (5)	56 (6)	2.10	3.24
Had difficulty finding a place to dispose of cloth or pad	791 (90)	28 (3)	22 (3)	37 (4)	3.34	9.99
Could not get the material I needed, like cotton cloth or sanitary pad	832 (95)	33 (4)	8 (1)	5 (1)	5.73	36.92
Had difficulty finding a suitable place to dry my menstrual cloth	749 (85)	49 (6)	27 (3)	53 (6)	2.66	5.76
Had difficulty finding someone to help me with bathing and other menstruation-related needs at onset	683 (78)	152 (17)	17 (2)	26 (3)	2.57	6.83
Had to stay separated at night from my normal bed	762 (87)	69 (8)	13 (1)	34 (4)	3.26	10.09
**Factor 3: Symptoms**						
Experienced pain in the hands or legs during menstruation	329 (37)	174 (20)	134 (15)	241 (27)	0.24	1.55
Experienced stomach pains during menstruation	340 (39)	179 (20)	105 (12)	254 (29)	0.28	1.57
**Factor 4: Menstrual Cycle Concerns**						
Worried that my cycle was irregular	798 (91)	26 (3)	10 (1)	44 (5)	3.47	10.67
Worried about ability to become pregnant because of problems with my menstrual cycle	849 (97)	12 (1)	7 (1)	10 (1)	6.55	44.00
Worried about my health because of problems with my menstrual cycle	790 (90)	44 (5)	12 (1)	32 (4)	3.65	12.57
**Factor 5: Bodily Concerns**						
Got wounds on inner thighs from cloth or belt	655 (75)	182 (21)	20 (2)	21 (2)	2.32	5.78
Worried that my cloth, napkin or my body smelled	756 (86)	42 (5)	42 (5)	38 (4)	2.73	6.28
Had trouble doing my work because of wound from cloth/pad	774 (88)	74 (8)	11 (1)	19 (2)	3.72	14.55
**Deleted Items**						
Avoided leaving the house during menstruation	470 (54)	53 (6)	162 (18)	193 (22)	0.49	1.50
Did not feel like eating during menstruation	471 (54)	227 (26)	77 (9)	103 (12)	1.10	0.06
Worried about needing other's support to get the cloth or sanitary pads I needed	688 (78)	140 (16)	25 (3)	25 (3)	2.52	6.35
Experienced headache during menstruation	693 (79)	114 (13)	33 (4)	38 (4)	2.41	5.10
Did not feel like interacting with others during menstruation	668 (76)	112 (13)	33 (4)	65 (7)	2.07	3.07
Experienced burning and irritation in urinary tract during menstruation	683 (78)	131 (15)	36 (4)	28 (3)	2.37	5.21
Got blood stains or leaks on my clothes	755 (86)	106 (12)	11 (1)	6 (1)	3.23	12.38
Experienced heavy bleeding	712 (81)	98 (11)	34 (4)	34 (4)	2.55	5.83
Felt like vomiting during menstruation	779 (89)	50 (6)	19 (2)	30 (3)	3.42	11.01
Had a lot of cleaning work to do because of menstruation	627 (71)	87 (10)	113 (13)	51 (6)	1.50	0.87
Had difficulty doing my regular work during menstruation	506 (58)	190 (22)	105 (12)	77 (9)	1.14	0.05

Table S3. Menstrual Insecurity Item frequencies among women in rural Orissa, India (*n* = 878) *(Continued)*.

	**Full Sample** (%)					
	Never	Sometimes	Often	Always	Skewness	Kurtosis
**Deleted Items** (Continued)						
Had difficulty fetching water for menstruation related needs	556 (63)	143 (16)	88 (10)	91 (10)	1.27	0.21
Had difficulty urinating while menstruating	627 (71)	147 (17)	50 (6)	54 (6)	1.86	2.44
Had difficulty walking during menstruation	508 (58)	230 (26)	53 (6)	87 (10)	1.34	0.67
Was forced to conform to a restriction that I do not believe in	729 (83)	117 (13)	9 (1)	23 (3)	3.12	10.36
Had people tease me because they knew I was menstruating	865 (99)	11 (1)	1 (0)	1 (0)	11.49	161.46
Worried about being treated as untouchable by others	832 (95)	26 (3)	6 (1)	14 (2)	5.49	31.11
Had to wear cloth that was not fully dry after washing *	847 (97)	26 (3)	4 (0)	0 (0)	6.08	40.36
Have a general feeling of tension at onset of menstruation	307 (35)	139 (16)	180 (21)	252 (29)	0.06	–1.60
Was prevented from going to certain places that I wanted to go	666 (76)	110 (13)	11 (1)	91 (10)	1.99	2.53
Worried that others knew I was menstruating	862 (98)	9 (1)	3 (0)	4 (0)	9.57	98.86

*1 participant missing

Table S4. Demographic characteristics of survey participants, overall and by random split halves, in rural Orissa, India ($N = 878$).

Characteristics	All		Sub-Sample N_1		Sub-Sample N_2		Chi-square/t-test	p-value
Number of Participants	**878**		**426**		**452**			
Former Intervention Community	436	49.7%	215	50.5%	221	48.9%	0.22	0.64
Age*	26.80	7.68	27.08	7.89	26.54	7.48	1.03	0.30
Hindu	866	98.6%	420	98.6%	446	98.7%	0.01	0.92
**Caste** [1]							11.98	0.04
Brahmin	26	3.0%	8	1.9%	18	4.0%		
General Caste	392	44.7%	206	48.5%	186	41.2%		
Scheduled Caste (SC)	158	18.0%	70	16.5%	88	19.5%		
Other Backward Caste (OBC)	283	32.3%	137	32.2%	146	32.4%		
Scheduled Tribe (ST)	5	0.6%	2	0.5%	3	0.7%		
Don't Know	12	1.4%	2	0.5%	10	2.2%		
**Education**							6.69	0.08
None	69	7.9%	35	8.2%	34	7.5%		
Some Primary	245	27.9%	133	31.2%	112	24.8%		
Some Secondary	492	56.0%	230	54.0%	262	58.0%		
Higher than Secondary	72	8.2%	28	6.6%	44	9.7%		
Possession of Below Poverty Line (BPL) Card [1]	549	62.6%	270	63.4%	279	61.9%	1.62	0.66
Have Children	447	50.9%	224	52.6%	223	49.3%	0.92	0.34
Number of Children *	1.01	1.26	1.69	1.28	1.62	1.21	0.66	0.51
No Current Illness [1]	776	88.6%	376	88.5%	399	88.5%	0.00	1.00
**Household Water and Sanitation Access**								
Functional Latrine in Household [1]	288	32.9%	143	34.3%	145	32.1%	0.22	0.89
Primary Drinking Water Source within Dwelling/Compound [2]	242	29.3%	126	31.0%	116	27.7%	2.41	0.30
Bathing Room in Household [1]	131	15.1%	67	15.8%	64	14.3%	0.39	0.53

Data are number and percent or mean (and standard deviation). [1] For *Caste*: 2 missing and 12 indicated don't know; For *BPL Card*: 1 missing; For *No Current Illness*: 2 missing; For *Social Support*: 2 missing; For *Bathing Room in Household*: 8 missing; For Mental Health Outcomes: 2 missing. [2] For *Primary Drinking Source*: data taken from census, 53 missing; For *Functional Latrine in Household*: data taken from census, 5 missing; * t-test statistic and p-value reported

Table S5. Participant menstruation practices, overall and by life stage, in rural Orissa, India (n = 878)

	**All**		**1. Unmarried (UM)**		**2. Recently Married (RM)**		**3. Married (M)**		**4. Over 49 (OW)**	
**Experiencing Monthly Menstruation**	878		338		222		308		10	
Absorbent Material Used for Menstruation [1]										
Reusable Cloth	611	69.7%	224	66.3%	112	50.5%	265	86.3%	10	100.0%
Disposable Pad	203	23.4%	83	24.6%	89	40.1%	31	10.1%	0	0.0%
Both Cloth and Pad	61	7.0%	31	9.2%	21	9.5%	9	32.9%	0	0.0%
Nothing	2	0.2%	0	0.0%	0	0.0%	2	0.7%	0	0.0%
**Frequency Changing Material on Heaviest Day**	2.51	1.05	2.58	1.00	2.63	0.91	2.30	0.89	3.90	4.18
Reuse Absorbent Material[1]										
Reuse	610	70.0%	226	67.3%	120	54.1%	255	83.9%	9	90.0%
Throw Away	262	30.0%	110	32.7%	102	45.9%	49	16.1%	1	10.0%
**Location for Changing Absorbent Material** [1]										
Household Toilet	25	2.9%	8	2.4%	6	2.7%	11	3.6%	0	0.0%
Bathroom	48	5.5%	13	3.9%	19	8.6%	15	4.9%	1	10.0%
In Toilet of Neighbor/Relative	3	0.3%	0	0.0%	1	0.8%	2	0.7%	0	0.0%
Private Room in Home	782	89.3%	312	92.3%	191	86.4%	271	88.3%	8	80.0%
Outside	16	1.8%	5	1.5%	4	1.8%	6	2.0%	1	10.0%
**Location for Disposing Absorbent Material** [2]										
Flush down Toilet/In Pit Latrine	4	1.5%	2	1.8%	1	1.0%	1	2.0%	0	0.0%
Rubbish Bin	9	3.4%	3	2.7%	5	5.0%	1	2.0%	0	0.0%
Pond/River/Stream	172	65.9%	78	70.9%	61	60.4%	32	65.3%	1	100.0%
Drain in Courtyard	3	1.1%	1	0.9%	1	1.0%	1	2.0%	0	0.0%
Throw Outside in Trash Pile	70	26.8%	24	21.8%	32	31.7%	14	28.6%	0	0.0%
Other	3	1.1%	2	1.8%	1	1.0%	0	0.0%	0	0.0%
**Location for Washing Absorbent Material** [3]										
Toilet Stall	23	3.8%	5	2.2%	7	5.8%	11	4.3%	0	0.0%
Bathroom	25	4.1%	6	2.7%	10	8.3%	8	3.1%	1	11.1%
Toilet of Neighbor/Relative	1	0.2%	0	0.0%	1	0.8%	0	0.0%	0	0.0%
Private Tube Well	68	11.1%	24	10.6%	19	15.8%	22	8.6%	3	33.3%
Public Tap Well	26	4.3%	10	4.4%	6	5.0%	10	3.9%	0	0.0%
Pond/River	434	71.1%	173	76.5%	68	56.7%	190	74.5%	3	33.3%
Courtyard/Yard	28	4.6%	7	3.1%	8	6.7%	12	4.7%	1	11.1%
Other	5	0.8%	1	0.4%	1	0.8%	2	0.8%	1	11.1%
**Location for Drying Absorbent Material** [3]										
Outside in Sun	487	79.8%	181	80.1%	95	79.2%	202	79.2%	9	100.0%
Outside not in Sun	58	9.5%	22	9.7%	7	5.8%	29	11.4%	0	0.0%
Inside	65	10.7%	23	10.2%	18	15.0%	24	9.4%	0	0.0%
**Location for Storing Absorbent Material** [3]										
With Clothes	4	0.7%	4	1.8%	0	0.0%	0	0.0%	0	0.0%
In Toilet Stall	5	0.8%	1	0.4%	1	0.8%	3	1.2%	0	0.0%
In Eaves of Roof	247	40.5%	71	31.4%	39	32.5%	130	51.0%	7	77.8%
Hidden in House	303	49.7%	131	58.0%	66	55.0%	104	40.8%	2	22.2%
Hidden outside House	49	8.0%	19	8.4%	12	10.0%	18	7.1%	0	0.0%
Other	2	0.3%	0	0.0%	2	1.7%	0	0.0%	0	0.0%

Data are number and percent or mean (and standard deviation). [1] For *Menstruation Material*: 1 missing (stage 3); For *Location Changing Absorbent Material*: 2 missing (stage 2 & stage 3); For *Reuse Absorbent Materials*: 4 missing (stage 1 & stage 3), 2 excluded (stage 3). [2] Limited to women that throw away absorbent material, 1 missing (stage 2). [3] Limited to women that reuse absorbent material

Table S6. Menstrual Insecurity item frequencies among women in rural Orissa, India, by random split half (n = 878)

	**Sub-Sample n_1 (n = 426)** (%)						**Sub-Sample n_2 (n = 452)** (%)					
	Never	Sometimes	Often	Always	Skew	Kurtosis	Never	Sometimes	Often	Always	Skew	Kurtosis
**Factor 1: Restrictions**												
Could not touch certain things, which created difficulties for me	137 (32)	132 (31)	63 (15)	94 (22)	0.38	−1.26	160 (35)	144 (32)	66 (15)	82 (18)	0.52	−1.05
Worry about not being able to participate in religious activities	116 (27)	122 (29)	62 (15)	126 (30)	0.13	−1.48	125 (28)	139 (31)	59 (13)	129 (29)	0.20	−1.44
**Factor 2: Management**												
Had difficulty finding a place to change menstrual materials (cloth or pad)	389 (91)	17 (4)	11 (3)	9 (2)	3.90	15.02	415 (92)	18 (4)	4 (1)	15 (3)	4.08	15.97
Had difficulty finding a place to wash cloth	346 (81)	25 (6)	21 (5)	34 (8)	2.14	3.07	371 (82)	26 (6)	19 (4)	36 (8)	2.22	3.44
Had difficulty finding a place to store menstrual cloth or pads	400 (94)	14 (3)	2 (0)	10 (2)	4.96	24.64	419 (93)	16 (4)	7 (2)	10 (2)	4.38	19.14
Experienced difficulty bathing during menstruation	334 (78)	46 (11)	19 (4)	27 (6)	2.19	3.64	342 (76)	57 (13)	24 (5)	29 (6)	2.02	2.94
Had difficulty finding a place to dispose of cloth or pad	388 (91)	10 (2)	10 (2)	18 (4)	3.47	10.76	403 (89)	18 (4)	12 (3)	19 (4)	3.25	9.45
Could not get the material I needed, like cotton cloth or sanitary pad	401 (94)	17 (4)	6 (1)	2 (0)	5.17	29.58	431 (95)	16 (4)	2 (0)	3 (1)	6.40	46.80
Had difficulty finding a suitable place to dry my menstrual cloth	358 (84)	26 (6)	13 (3)	29 (7)	2.51	4.94	391 (87)	23 (5)	14 (3)	24 (5)	2.83	6.75
Had difficulty finding someone to help me with bathing and other menstruation-related needs at onset	319 (75)	86 (20)	10 (2)	11 (3)	2.34	5.81	364 (81)	66 (15)	7 (2)	15 (3)	2.80	7.95
Had to stay separated at night from my normal bed *	372 (87)	36 (8)	9 (2)	9 (2)	3.46	12.31	390 (86)	33 (7)	4 (1)	25 (6)	3.04	8.20
**Factor 3: Symptoms**												
Experienced pain in the hands or legs during menstruation	162 (38)	77 (18)	72 (17)	115 (27)	0.22	−1.57	167 (37)	97 (21)	62 (14)	126 (28)	0.26	−1.54
Experienced stomach pains during menstruation	167 (39)	85 (20)	57 (13)	117 (27)	0.30	−1.54	173 (38)	94 (21)	48 (11)	137 (30)	0.27	−1.59
**Factor 4: Menstrual Cycle Concerns**												
Worried that my cycle was irregular	389 (91)	9 (2)	4 (1)	24 (6)	3.41	10.07	409 (90)	17 (4)	6 (1)	20 (4)	3.54	11.40
Worried about ability to become pregnant because of problems with my menstrual cycle	412 (97)	5 (1)	3 (1)	6 (1)	6.38	41.21	437 (97)	7 (2)	4 (1)	4 (1)	6.70	47.10
Worried about my health because of problems with my menstrual cycle	381 (89)	24 (6)	3 (1)	18 (4)	3.59	12.08	409 (90)	20 (4)	9 (2)	14 (3)	3.71	13.17

Table S7. Menstrual Insecurity item frequencies among women in rural Orissa, India, by random split half (n = 878) *(Continued)*.

	**Sub-Sample n1 (*n* = 426)** (%)						**Sub-Sample n2 (*n* = 452)** (%)					
	Never	Sometimes	Often	Always	Skew	Kurtosis	Never	Sometimes	Often	Always	Skew	Kurtosis
**Factor 5: Bodily Concerns**												
Got wounds on inner thighs from cloth or belt	318 (75)	85 (20)	10 (2)	13 (3)	2.34	5.62	337 (75)	97 (21)	10 (2)	8 (2)	2.26	5.77
Worried that my cloth, napkin or my body smelled	366 (86)	21 (5)	21 (5)	18 (4)	2.72	6.25	390 (86)	21 (5)	21 (5)	20 (4)	2.75	6.39
Had trouble doing my work because of wound from cloth/pad	373 (88)	37 (9)	6 (1)	10 (2)	3.60	13.42	401 (89)	37 (8)	5 (1)	9 (2)	3.87	15.95
**Deleted Items**												
Avoided leaving the house during menstruation	237 (56)	25 (6)	75 (18)	89 (21)	0.57	–1.42	233 (52)	28 (6)	87 (19)	104 (23)	0.42	–1.56
Did not feel like eating during menstruation	236 (55)	109 (26)	35 (8)	46 (11)	1.18	0.17	235 (52)	118 (26)	42 (9)	57 (13)	1.02	–0.25
Worried about needing other's support to get the cloth or sanitary pads I needed	327 (77)	70 (16)	16 (4)	13 (3)	2.34	5.26	361 (80)	70 (15)	9 (2)	12 (3)	2.73	7.75
Experienced headache during menstruation	341 (80)	52 (12)	13 (3)	20 (5)	2.51	5.58	352 (78)	62 (14)	20 (4)	18 (4)	2.32	4.73
Did not feel like interacting with others during menstruation	327 (77)	53 (12)	18 (4)	28 (7)	2.12	3.39	341 (75)	59 (13)	15 (3)	37 (8)	2.02	2.83
Experienced burning and irritation in urinary tract during menstruation	336 (79)	58 (14)	17 (4)	15 (4)	2.43	5.43	347 (77)	73 (16)	19 (4)	13 (3)	2.31	5.04
Got blood stains or leaks on my clothes	368 (86)	49 (12)	6 (1)	3 (1)	3.30	12.73	387 (86)	57 (13)	5 (1)	3 (1)	3.18	12.85
Experienced heavy bleeding	352 (83)	47 (11)	16 (4)	11 (3)	2.75	7.27	360 (80)	51 (11)	18 (4)	23 (5)	2.38	4.72
Felt like vomiting during menstruation	379 (89)	24 (6)	7 (2)	16 (4)	3.47	11.36	400 (88)	26 (6)	12 (3)	14 (3)	3.37	10.78
Had a lot of cleaning work to do because of menstruation	307 (72)	42 (10)	56 (13)	21 (5)	1.54	1.00	320 (71)	45 (10)	57 (13)	30 (7)	1.47	0.76
Had difficulty doing my regular work during menstruation	251 (59)	85 (20)	56 (13)	34 (8)	1.14	0.05	255 (56)	105 (23)	49 (11)	43 (10)	1.14	0.07
Had difficulty fetching water for menstruation related needs	270 (63)	68 (16)	50 (12)	38 (9)	1.26	0.22	286 (63)	75 (17)	38 (8)	53 (12)	1.28	0.19
Had difficulty urinating while menstruating	318 (75)	65 (15)	20 (5)	23 (5)	2.08	3.44	309 (68)	82 (18)	30 (7)	31 (7)	1.68	1.75
Had difficulty walking during menstruation	252 (59)	109 (26)	26 (6)	39 (9)	1.39	0.86	256 (57)	121 (27)	27 (6)	48 (11)	1.29	0.52
Was forced to conform to a restriction that I do not believe in	352 (83)	61 (14)	5 (1)	8 (2)	3.07	10.59	377 (83)	56 (12)	4 (1)	15 (3)	3.13	9.95
Had people tease me because they knew I was menstruating	418 (98)	6 (1)	1 (0)	1 (0)	10.12	118.61	447 (99)	5 (1)	0 (0)	0 (0)	9.38	86.38
Worried about being treated as untouchable by others	407 (96)	12 (3)	3 (1)	4 (1)	6.19	41.38	425 (94)	14 (3)	3 (1)	10 (2)	4.97	24.85
Had to wear cloth that was not fully dry after washing*[1]	408 (96)	18 (4)	0 (0)	0 (0)	4.57	18.95	439 (97)	8 (2)	4 (1)	0 (0)	7.04	52.21
Have a general feeling of tension at onset of menstruation	149 (35)	72 (17)	87 (20)	118 (28)	0.09	–1.58	158 (35)	67 (15)	93 (21)	134 (30)	0.03	–1.62
Was prevented from going to certain places that I wanted to go	320 (75)	59 (14)	6 (1)	41 (10)	2.02	2.75	346 (77)	51 (11)	5 (1)	50 (11)	1.97	2.37
Worried that others knew I was menstruating	415 (97)	8 (2)	1 (0)	2 (0)	8.66	84.61	447 (99)	1 (0)	2 (0)	2 (0)	10.66	117.34

*Chi-square test p-value < 0.05; [1] 1 participant missing (Sub-sample n2 = 451)

Table S8. Removed scale items with reasons for removal.

Item No.	**Item**	**Reason**
**M20**	Got blood stains or leaks on my clothes	Cross-loaded on four factors, lowest loading values below 0.6
**M18**	Experienced burning and irritation in urinary tract during menstruation	Cross-loaded on four factors, lowest loading values below 0.6
**M45**	Have a general feeling of tension at onset of menstruation	Cross-loaded on four factors, lowest loading values below 0.6
**M36**	Had people tease me because they knew I was menstruating	Highly correlated with M47, which loaded strongly into factor
**M46**	Was prevented from going to certain places that I wanted to go	Cross-loaded on four factors, lowest loading values below 0.6
**M10**	Avoided leaving the house during menstruation	All loading values less than 0.3
**M31**	Had difficulty fetching water for menstruation related needs	Cross-loaded on four factors, lowest loading values below 0.6
**M16**	Experienced headache during menstruation	Cross-loaded on three factors, lowest loading values below 0.6
**M17**	Did not feel like interacting with others during menstruation	Cross-loaded on three factors, lowest loading values below 0.6
**M15**	Worried about needing other's support to get the cloth or sanitary pads I needed	Cross-loaded on three factors, lowest loading values below 0.6
**M30**	Had difficulty doing my regular work during menstruation	Cross-loaded on three factors, lowest loading values below 0.6
**M34**	Had difficulty walking during menstruation	Cross-loaded on three factors, lowest loading values below 0.6
**M22**	Experienced heavy bleeding	Cross-loaded on two factors, lowest loading values below 0.6
**M12**	Did not feel like eating during menstruation	Cross-loaded on two factors, lowest loading values below 0.6
**M26**	Felt like vomiting during menstruation	Cross-loaded on two factors, lowest loading values below 0.6
**M32**	Had difficulty urinating while menstruating	Cross-loaded on two factors, all items loaded on one factor above 0.6, lowest loading value above 0.6
**M35**	Was forced to conform to a restriction that I do not believe in	Cross-loaded on three factors, lowest loading values below 0.6
**M27**	Had a lot of cleaning work to do because of menstruation	Cross-loaded on two factors, only item loading below 0.6
**M49**	Worried that others knew I was menstruating	Cross-loaded on three factors, all items loaded on one factor above 0.6, lowest loading value above 0.6
**M43**	Had to wear cloth that was not fully dry after washing	Cross-loaded on four factors
**M42**	Worried about being treated as untouchable by others	Cross-loaded on three factors

Table S9. Items used for validation of scale.

Items	All		1. Unmarried (UM)		2. Recently Married (RM)		3. Married (M)		4. Over 49 (OW)	
Number of Participants	878		338	38.5%	222	25.3%	308	35.1%	10	1.1%
**Travel Outside Village [1]**										
Alone	143	16.4%	90	26.6%	3	1.4%	48	15.7%	2	20.0%
With Someone Else	509	58.2%	203	60.1%	140	63.3%	160	52.5%	6	60.0%
Not at All	222	25.4%	45	13.3%	78	35.3%	97	31.8%	2	20.0%
**Travel to Defecate [1]**										
Alone	662	75.7%	229	67.8%	166	75.1%	258	84.6%	9	90.0%
With Someone Else	208	23.8%	109	32.2%	52	23.5%	46	15.1%	1	10.0%
Not at All	4	0.5%	0	0.0%	3	1.4%	1	0.3%	0	0.0%
**Travel to Water Source [2]**										
Alone	721	82.4%	303	89.6%	134	60.6%	274	89.5%	10	100.0%
With Someone Else	20	2.3%	8	2.4%	7	3.2%	5	1.6%	0	0.0%
Not at All	134	15.3%	27	8.0%	80	36.2%	27	8.8%	0	0.0%
**Difficulty Finding Private Place to Urinate**										
Never	418	47.6%	148	43.8%	112	50.5%	153	49.7%	5	50.0%
Sometimes	199	22.7%	76	22.5%	44	19.8%	75	24.4%	4	40.0%
Often	134	15.3%	61	18.0%	23	10.4%	50	16.2%	0	0.0%
Always	127	14.5%	53	15.7%	43	19.4%	30	9.7%	1	10.0%
Bathing Room in Household [3]	131	15.1%	26	7.7%	66	29.9%	38	12.5%	1	10.0%
Abnormal Vaginal Discharge [4]	237	27.1%	102	30.2%	59	26.6%	72	23.5%	4	40.0%
Burning or Itching when Urinating [4]	106	12.1%	28	8.3%	31	14.0%	45	14.7%	2	20.0%
Burning or Itching in Vaginal Area [4]	107	12.2%	34	10.1%	30	13.5%	42	13.7%	1	10.0%
Reuse Absorbent Material [5]	610	70.0%	226	67.3%	120	54.1%	255	83.9%	9	90.0%
**Currently Experience Periods**										
Regular	750	85.4%	302	89.3%	189	85.1%	254	82.5%	5	50.0%
Irregular	128	14.6%	36	10.7%	33	14.9%	54	17.5%	5	50.0%

[1] 4 missing (stage 2 & stage 3); [2] 3 missing (stage 2 & stage 3); [3] For *Bathing Room in Household:* 8 missing (stage 1, stage2, & stage3); [4] Reported for occurrence in past two weeks, 2 missing (stage 3); [5] 6 missing (stage 1 & stage 3)

Table S10. Average weighted Menstrual Insecurity sub-scale scores by life stage-, health-, and sanitation-related items (N = 878).

Characteristics	Menstrual Insecurity	Factor 1: Restrictions	Factor 2: Management	Factor 3: Symptoms	Factor 4: Menstrual Cycle Concerns	Factor 5: Bodily Concerns
All	1.49 (0.36)	2.34 (1.07)	1.25 (0.45)	2.32 (1.13)	1.15 (0.46)	1.23 (0.49)
**Life Stage**						
Unmarried Women (Ref.)	1.48 (0.37)	2.08 (0.99)	1.26 (0.45)	2.45 (1.12)	1.15 (0.43)	1.24 (0.51)
Recently Married Women	1.52 (0.40)	2.39 (1.06) *	1.28 (0.51)	2.40 (1.17)	1.18 (0.51)	1.20 (0.49)
Married Women	1.48 (0.33)	2.56 (1.11) *	1.22 (0.41)	2.13 (1.06) *	1.13 (0.45)	1.24 (0.46)
Older Women	1.48 (0.23)	2.73 (1.17) *	1.16 (0.28)	1.96 (1.23)	1.27 (0.65)	1.25 (0.48)
**Ownership of Functional Latrine**						
Owns (Ref.)	1.41 (0.30)	2.19 (1.05)	1.14 (0.32)	2.28 (1.10)	1.13 (0.43)	1.18 (0.42)
Does Not Own	1.53 (0.38) *	2.40 (1.08) *	1.31 (0.50) *	2.34 (1.14)	1.16 (0.47)	1.26 (0.52) *
**Enclosed Bathing Area**						
Owns (Ref.)	1.36 (0.27)	2.15 (1.04)	1.07 (0.21)	2.31 (1.14)	1.09 (0.36)	1.14 (0.39)
Does Not Own	1.51 (0.37) *	2.36 (1.08) *	1.28 (0.48) *	2.32 (1.12)	1.16 (0.47)	1.25 (0.51) *
**Water Source** [1]						
Inside Compound (Ref.)	1.42 (0.30)	2.30 (1.06)	1.11 (0.27)	2.38 (1.13)	1.11 (0.39)	1.21 (0.47)
Outside Compound	1.52 (0.38) *	2.36 (1.10)	1.30 (0.49) *	2.30 (1.12)	1.16 (0.48)	1.24 (0.51)
**Menstruation Materials** [1]						
Reusable Cloth (Ref.)	1.50 (0.37)	2.37 (1.09)	1.26 (0.46)	2.27 (1.11)	1.17 (0.49)	1.27 (0.53)
Disposable Pad	1.43 (0.31) *	2.22 (1.03)	1.17 (0.36) *	2.44 (1.18)	1.09 (0.31) *	1.14 (0.37) *
Both Pad and Cloth	1.57 (0.45)	2.35 (1.11)	1.42 (0.61)	2.43 (1.09)	1.21 (0.57)	1.16 (0.40) *
Nothing	1.07 (0.09)	1.00 (0.00) *	1.00 (0.00) *	1.30 (0.43)	1.17 (0.24)	1.00 (0.00) *
**Tension at Menstruation Onset**						
Never (Ref.)	1.33 (0.27)	2.01 (0.96)	1.12 (0.29)	2.02 (1.06)	1.13 (0.39)	1.08 (0.20)
Sometimes	1.50 (0.34) *	2.18 (0.88)	1.29 (0.46) *	2.36 (1.08) *	1.23 (0.55)	1.20 (0.35) *
Often	1.49 (0.29) *	2.37 (0.93) *	1.23 (0.35) *	2.25 (1.01) *	1.08 (0.31)	1.37 (0.50) *
Always	1.68 (0.43) *	2.79 (1.23) *	1.41 (0.60) *	2.72 (1.18) *	1.18 (0.56)	1.33 (0.70) *
**Currently Ill** [1]						
Yes (Ref.)	1.54 (0.36)	2.32 (1.06)	1.35 (0.54)	2.36 (1.13)	1.22 (0.55)	1.21 (0.39)
No	1.48 (0.36)	2.34 (1.08)	1.24 (0.44)	2.32 (1.13)	1.14 (0.45)	1.23 (0.50)

[1] For *Enclosed Bathing Area:* 8 missing; For *Water Source*: 53 participants missing; For *Currently Ill*: 2 participants missing. [2] For *Menstruation Materials*: 1 participant missing, 611 Reusable Cloth, 203 Disposable Pad, 61 Both, 2 None; * p-value < 0.05
